# INTEGRATED CARE POLICY AND PRACTICE IN THE US: THE SCENARIO OF AGING IN CHINATOWN

**DOI:** 10.1093/geroni/igac059.1993

**Published:** 2022-12-20

**Authors:** Yuanyuan Hu, Qingwen Xu

**Affiliations:** New York University, New York, New York, United States; New York University, New York, New York, United States

## Abstract

**Background:**

Older Chinese adults, the fastest-growing population among older immigrants, experience multiple barriers to access quality physical and behavioral health care, including low English proficiency, low health literacy, and segregation between health care and social care sectors (Tsoh et al., 2016). While integrated care attempts to address these issues, there is still a lack of culturally sensitive integrated care practices to address the needs of older Chinese immigrants.

**Methods:**

This article reviews the definition and history of integrated care policies in the U.S., and compares four integrated care models on the service user and community levels, including the Chronic Care Model (CCM), Program of All-Inclusive Care for the Elderly (PACE), Patient Navigation Model, and Delivery System Reform Incentive Payment (DSRIP) Program.

**Results:**

Taking the community-dwelling older Chinese immigrants as the context, this article discusses factors that are essential to this group of older adults and proposes a framework to integrate social determinants of health in the development of integrated care practice with the infusion of cultural values and norms.

**Conclusion:**

Integrated care for older immigrants asks for a complicated mass reconstruction of current care systems. We propose an innovative framework that fully takes advantage of CBO’s capacity in providing culturally appropriate services is proactive and preventive in nature by addressing social determinants of health directly, recognizes the role of family and community in older immigrants’ life and aging process, and provide equal attention to the older adults’ needs in health, mental health, and elderly care.

